# Role of Nasal Endoscopy in Chronic Osteomyelitis of Maxilla and Zygoma: A Case Report

**DOI:** 10.1155/2011/802964

**Published:** 2011-11-01

**Authors:** J. S. Arunkumar, Ashok S. Naik, K. C. Prasad, S. G. Santhosh

**Affiliations:** Department of ENT, SDM College of Medical Sciences & Hospital, Karnataka, Dharwad, India

## Abstract

Osteomyelitis of the jaws was relatively common before the era of antibiotic therapy and preventive and restorative dentistry. Osteomyelitis is an acute or chronic inflammatory process that can involve cortical and trabecular aspects of bone or bone marrow. Cranial bones are infrequently involved, but spreading of inflammation with involvement of surrounding structures represents important risk, such as cerebral abscess, encephalitis, or meningitis. The mandible is more commonly involved than the maxilla. Dentogenic infections cause 38% of mandibular and 25% of maxillary involvement. Involvement of zygoma is very rare. Factors like viral fevers, malnutrition, malaria, anemia,tobacco chewing, immunodeficiency, osteopetrosis, Paget's disease of bone, and florid cemento-osseous dysplasia (FCOD) result in decreased vascularity of the affected bone, predisposing to the development of osteomyelitis. We present a case of osteomyelitis of left maxilla and zygoma with oroantral fistula in an immunocompetent adult male caused by dentogenic infection. The complete resolution of infection was gained with surgical treatment using nasal endoscope and antibiotic therapy. The aims of this paper are to illustrate diagnostic patterns, to report radiographic findings and surgical treatment using nasal endoscope in a case of osteomyelitis of maxilla and zygoma. The prognosis and cosmetic results are discussed.

## 1. Introduction

Osteomyelitis of the facial bones is a rare condition. Osteomyelitis is an acute or chronic inflammatory process that can involve cortical and trabecular aspects of bone or bone marrow. Cranial bones are infrequently involved. Osteomyelitis of zygoma seldom occurs because the zygoma receives a relatively sufficient supply of blood [1]. Furthermore, antibiotic therapy usually results in high rate of cure. A few cases of osteomyelitis of the facial bones have been reported and generally involve lesions of the maxilla or mandible that developed after trauma or dental infections [2]. Osteomyelitis of maxilla was originally described by Rees in 1847. Osteomyelitis of zygoma is extremely rare; only few cases of primary tuberculosis of zygomatic bone are reported in literature [3]. Osteomyelitis represents an inflammation of the medullary cavity, Haversian system, and adjacent cortex bone. In full-blown cases, there is necrosis of bone and deposition of calcium. Predisposing factors which contribute to the development of osteomyelitis include viral fevers, malnutrition, malaria, anemia, tobacco chewing, immunodeficiency, and so forth [4]. Most of the infections are as a result of polymicrobial oral flora which includes facultative streptococci, *Bacteroides spp.*, *Peptostreptococcus*, and *Peptococcus* [4]. The complete resolution of infection occurs with a combination of surgical treatment and antibiotic therapy. Operative interventions include procedures such as sequestrectomy, decortications, removal of nonviable bone (i.e., mandibulectomy or maxillectomy) dental extractions, and fracture fixation. 

## 2. Case Report

**Figure 1:**
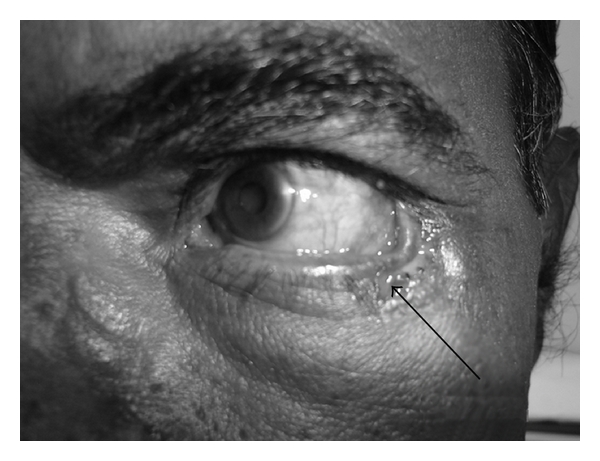
Preoperative picture showing discharging ulcer over left cheek.

**Figure 2:**
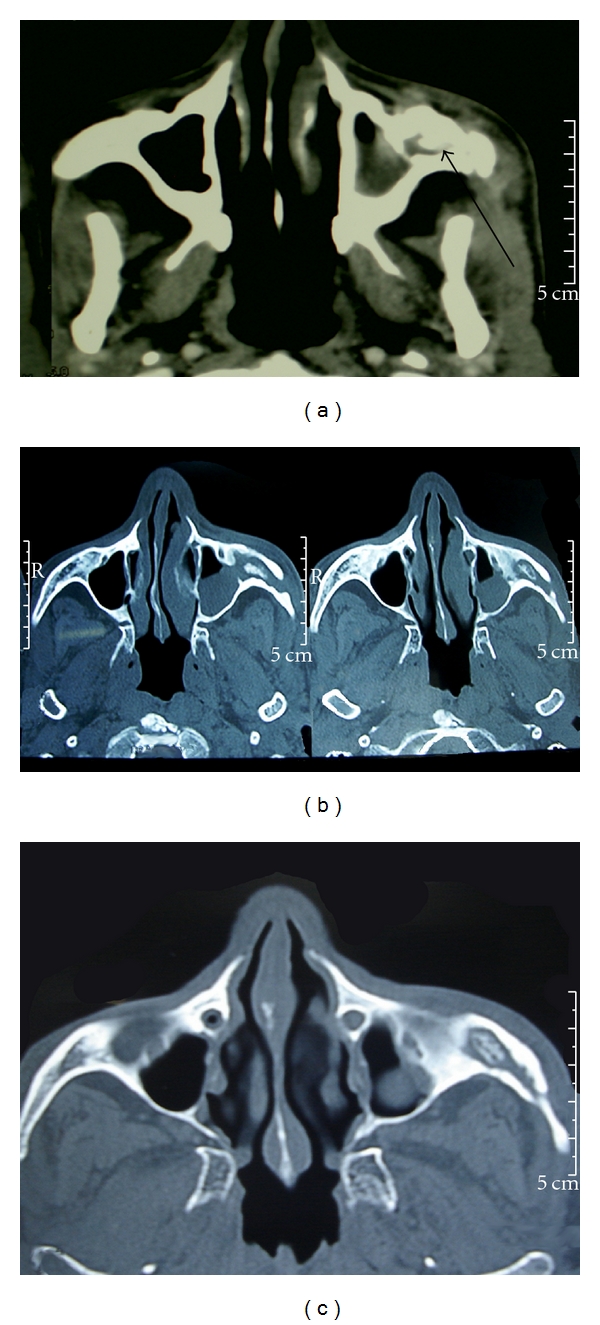
CT scan showing osteomyelitis of left maxilla and zygoma.

**Figure 3:**
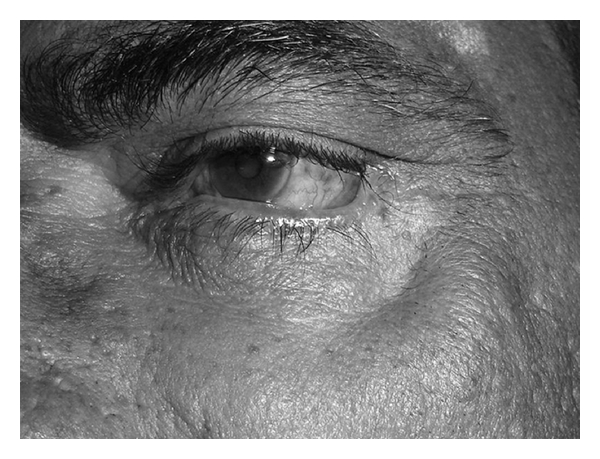
Postoperative picture demonstrating healed ulcer over left cheek.

A 54-year-old male patient presented to our department with discharging ulcer and swelling over the left cheek since 4-5 months. It started with toothache for which he took treatment from local doctor which did not respond. Later, he noticed nasal regurgitation of fluids and pain over the cheek on left side. On examination, his general physical examination was normal. Local examination revealed swelling of left zygoma with discharging sinus just below the left eyelid at the outer canthus of the left eye, with sprouting granulation tissue with thick pus and inflamed and tender surrounding area with mild ectropion (Figure 1). On intraoral examination, there was oroantral fistula at the area of second molar on left side. Nose, throat, and neck examination was normal. Provisional diagnosis of chronic osteomyelitis of maxilla and zygoma with oroantral fistula on left side was done. Dental opinion was taken. Dental caries and periodontitis in relation to upper left canine, 2nd premolar, and molar were involved. Extraction of the above teeth was done under local anaesthesia. Pus from the sinus was sent for culture and sensitivity which revealed coagulase negative staphylococci antibiotics given based on this report. CT (computed tomographic) scan of paranasal sinus with contrast was done which showed complete destruction of left maxillary alveolar process, anterior portion of hard palate, left zygoma and proximal 1/3rd of left zygomatic arch with oroantral fistula, and left maxillary sinusitis (Figures 2(a), 2(b), and 2(c)). Patient was started with oral ofloxacin 200 mg twice daily and analgesics for one week. The discharge from the sinus and pain reduced. Patient was operated electively under general anaesthesia. With all aseptic precautions, incision was taken about 3 mm below the lower eye lid margin extending up to 1 cm beyond the outer canthus of left eye. The fistula site was included in the incision. Incision was deepened up to the periosteum and fistula tract traced by widening the area using microdrill. Fistula tract and sequestrum measuring about 1.5 × 2 cms was removed. Nasal endoscope both, 0° and 30°, was used to visualize the maxillary sinus and the cavity of size 2 × 2 cms formed in the zygoma through the surgical incision taken below the left lower eye lid margin. All the hypertrophied mucosa and unhealthy bone were removed with a curette. Oroantral fistula in the left posterior alveolar process was closed with a buccal flap using 3-0 vicryl. Inferior meatal antrostomy was done; ribbon gauze pack soaked in antibiotic solution was kept in the maxillary sinus. The wound closed after saucerization of the bone with a microdrill. Pack was removed after 48 hrs; patient was given intravenous amoxicillin and clavulanic acid 1.2 gm twice daily along with analgesics for one week. The tissue was sent for histopathological examination; section studied showed necrotic osseous tissue with diffuse mixed inflammatory cells consisting of lymphocytes, plasma cells, and plenty of neutrophils suggestive of chronic Osteomyelitis. Post-op followup of one year showed no evidence of recurrence with minimal depression and asymptomatic mild ectropion (Figure 3).

## 3. Discussion

Osteomyelitis is an acute or chronic inflammatory process that can involve cortical and trabecular aspects of bone or bone marrow. Osteomyelitis represents an inflammation of the medullary cavity, Haversian system, and adjacent cortex bone. In full-blown cases, there is necrosis of bone and deposition of calcium. Paget 's disease of bone and florid cemento-osseous dysplasia (FCOD) result in decreased vascularity of the affected bone, predisposing to the development of osteomyelitis [5, 6]. Cranial bones are infrequently involved.

Hudson (1993) wrote that “Acute Osteomyelitis of the jaws may manifest itself with fever, malaise, facial cellulits [sic], trismus and significant leukocytosis. Osteomyelitis of the jaws of a chronic nature has findings consistent with swelling, pain, purulence, intraoral or extraoral draining fistulae, nonhealing bony and soft tissue wounds” [4]. Computerized tomography gives a more definitive picture of the calcified tissue involvement, especially with regard to disruption of the cortical plates. Diagnosis is based on the presence of painful sequestra and suppurative areas of tooth-bearing jaw bone not responding to debridement and conservative therapy.

The goal of definitive therapy is to attenuate and eradicate the proliferating pathogenic microorganisms and to support healing. 

The treatment guideline for osteomyelitis of jaws includes [4]:

- disrupt the infectious foci,
- debride any foreign body, necrotic tissue, or sequestra,
- culture and identify pathogens for definitive antibiotic treatment,
- drain and irrigate the region,
- stabilize calcified tissue regionally,
- adjunctive treatment to enhance microvascular reperfusion (usually reserved for refractory forms only):
  1. trephination,
  2. decortications,
  3. vascular flaps,
  4. hyperbaric oxygen therapy,
- reconstruction as necessary following resolution of the infection.

## 4. Conclusion

Osteomyelitis occurs when a bone becomes infected; though osteomyelitis most often occurs in the bones of the limbs, spine, and pelvis, it can also affect the jaw. Osteomyelitis in the jaw is a rare condition that once had been thought incurable. It can present in either acute or chronic forms. Early diagnosis and prompt treatment will prevent complications and deformities due to destruction of the bones. In our case, nasal endoscope was used to clear the disease inside the maxillary sinus and also to clear the dead and unhealthy bone over the zygoma. Advantage of using nasal endoscope was small incision and complete disease clearance with minimal depression and asymptomatic mild ectropion.

## References

[1] Kosaka Y, Yanai A, Katayama M (1996). Idiopathic osteomyelitis of the zygoma. *Plastic and Reconstructive Surgery*.

[2] Wolfowitz BL (1971). Osteomyelitis of the maxilla. *South African Medical Journal*.

[3] Chakravarti A , Dhawan R, Sahni JK (2005). Tubercular Osteomyelitis of zygomatic bone. *Indian Journal of Tuberculosis*.

[4] Hudson JW (1993). Osteomyelitis of the jaws: a 50-year perspective. *Journal of Oral and Maxillofacial Surgery*.

[5] Ogutcen-Toller M, Tek M, Sener I, Bereket C, Inal S, Özden B (2010). Intractable bimaxillary Osteomyelitis in osteopetrosis: review of the literature and current therapy. *Journal of Oral and Maxillofacial Surgery*.

[6] Chattopadhyay P, Kundu AK, Saha AK, Karthak RO (2008). Mandibular osteomyelitis and multiple skeletal complications in Albers-Schönberg disease. *Singapore Medical Journal*.

